# Severity of Downgaze Palsy in the Context of Disease Duration Could Estimate Survival Duration in Patients With Progressive Supranuclear Palsy

**DOI:** 10.3389/fneur.2021.736784

**Published:** 2021-09-28

**Authors:** Tao Xie, Carlen A. Yuen, Wenjun Kang, Mahesh Padmanaban, Timothy C. Hain, Jeffrey Nichols

**Affiliations:** ^1^Department of Neurology, University of Chicago Medicine, Chicago, IL, United States; ^2^Department of Neurology, Columbia University Medical Center, New York, NY, United States; ^3^Center of Research Informatics, University of Chicago, Chicago, IL, United States; ^4^Chicago Dizziness and Hearing, Northwestern University, Chicago, IL, United States; ^5^Department of Ophthalmology, University of Chicago Medicine, Chicago, IL, United States

**Keywords:** PSP, downgaze palsy, disease duration, survival duration, factors, severity

## Abstract

It is an unmet need to estimate survival duration for patients with progressive supranuclear palsy (PSP). The objective of this study was to identify factors associated with the survival duration in patients with PSP. We followed up 23 patients with probable PSP-RS (Richardson syndrome) or PSP-P (parkinsonism) in our PSP center until death from 2011 to 2019. We prospectively and quantitatively rated their downgaze palsy whenever first noticed in our clinic. This was utilized along with the disease duration, motor function, medication use for parkinsonism, sex, age at onset of PSP, comorbid pulmonary and cardiovascular diseases, and the total survival duration from the onset of PSP to death for prediction analysis. A well-fitted linear regression model and a multivariant Cox model were applied to identify predicting factors for total survival duration. All patients had the specific hummingbird sign on brain MRI for PSP when downgaze palsy was documented. We found that the severity of downgaze palsy and the disease duration at the assessment were consistently correlated with the total survival duration in both models. The total survival duration could be further estimated by a formed regression equation. We conclude that severity and time to develop downgaze palsy could help to estimate the total survival duration in patients with probable PSP-RS and PSP-P, the major forms of PSP, which has significant clinical applications in clinical counseling and trial enrollment.

## Introduction

Downgaze palsy in the first year of disease onset is a very specific feature of PSP-RS (progressive supranuclear palsy, Richardson syndrome) (1). Unlike upgaze, downgaze is not affected by age (2). Downgaze is mediated by oculomotor nuclei in the midbrain, with supranuclear input from the frontal and parietal cortex (3) and affected by damage along the path of the disease progression from the globus pallidus interna (GPi), subthalamic nucleus (STN), and substantia nigra (SN) of the midbrain (4). Hence, downgaze palsy can possibly mirror the disease progression in PSP, at least for PSP-RS and PSP-P (-parkinsonism). In our previous autopsy study, earlier onset of downgaze palsy (or shorter latency between the disease onset and the occurrence of downgaze palsy) was associated with a shorter survival duration of PSP (-RS, or -P) (5). This result is consistent with the observation that patients with PSP-RS (with earlier occurrence of downgaze palsy) have a worse prognosis and shorter survival duration than PSP-P (with later occurrence or even no occurrence of downgaze palsy before the death) (6). However, identifying the onset of downgaze palsy as soon as it occurs can be unrealistic in clinical practice as the patient may not closely follow up, or be seen in the same clinic. We hypothesized that the combination of the disease duration when downgaze palsy was first identified (not necessarily at the onset) with the severity of downgaze palsy might predict prognosis in clinical practice, regardless of when the downgaze palsy first occurs or when the patient is first seen. We suspect that severity and time to develop downgaze palsy could correlate or predict the survival. The potential roles of other factors in the survival duration could also be assessed, such as the sex, age at onset (AO) of PSP, medication use for motor symptoms (symptomatic use for parkinsonism), the severity of the motor symptoms, and the presence of pulmonary and cardiovascular diseases (as aspiration pneumonia and its complications are the common causes of death in PSP) (7, 8).

To date, studies have rarely been conducted on predicting specific survival duration in PSP patients, which is an important unmet need for clinical counseling, family planning, and clinical trial enrollment. Here, we present our study on factors affecting survival and form a preliminary equation to estimate the total survival duration for patients with probable PSP (-RS or -P) and downgaze palsy.

## Methods

### Standard Protocol Approvals and Data Source

This study was approved by the Institutional Review Board (IRB) at the University of Chicago Medicine. Consent was obtained according to the Declaration of Helsinki. We identified patients with probable PSP-RS or PSP-P based on the MDS (International Movement Disorder Society) task force criteria (9). All patients were followed up by a movement disorder specialist (TX) in our PSP Center before they died, between 2011 and 2019. We collected information on sex, AO of PSP, disease duration when the downgaze palsy was first quantitatively documented (regardless of when they came to our clinic, and not necessarily the onset of the downgaze palsy), and the severity rated at that time, the UPDRS-III (Unified Parkinson's Disease Rating Sale, motor section) scores, medication use then or following a visit for parkinsonism, cardiovascular diseases or risk factors (hypertension, diabetes, dyslipidemia, congestive heart failure, atrial fibrillation, coronary artery disease), and pulmonary diseases (chronic obstructive pulmonary disease, asthma, pneumonia). Downgaze palsy severity was prospectively quantified by percentage of the limit in downgaze amplitude, with 0% limit as normal without downgaze palsy, 10% limit as the least palsy, and 100% as the worst or complete palsy. Patients who died from causes unrelated to PSP, such as cancers, stroke, and motor vehicle accident, were excluded.

### Measurement of Downgaze Palsy

**Figure 1:**
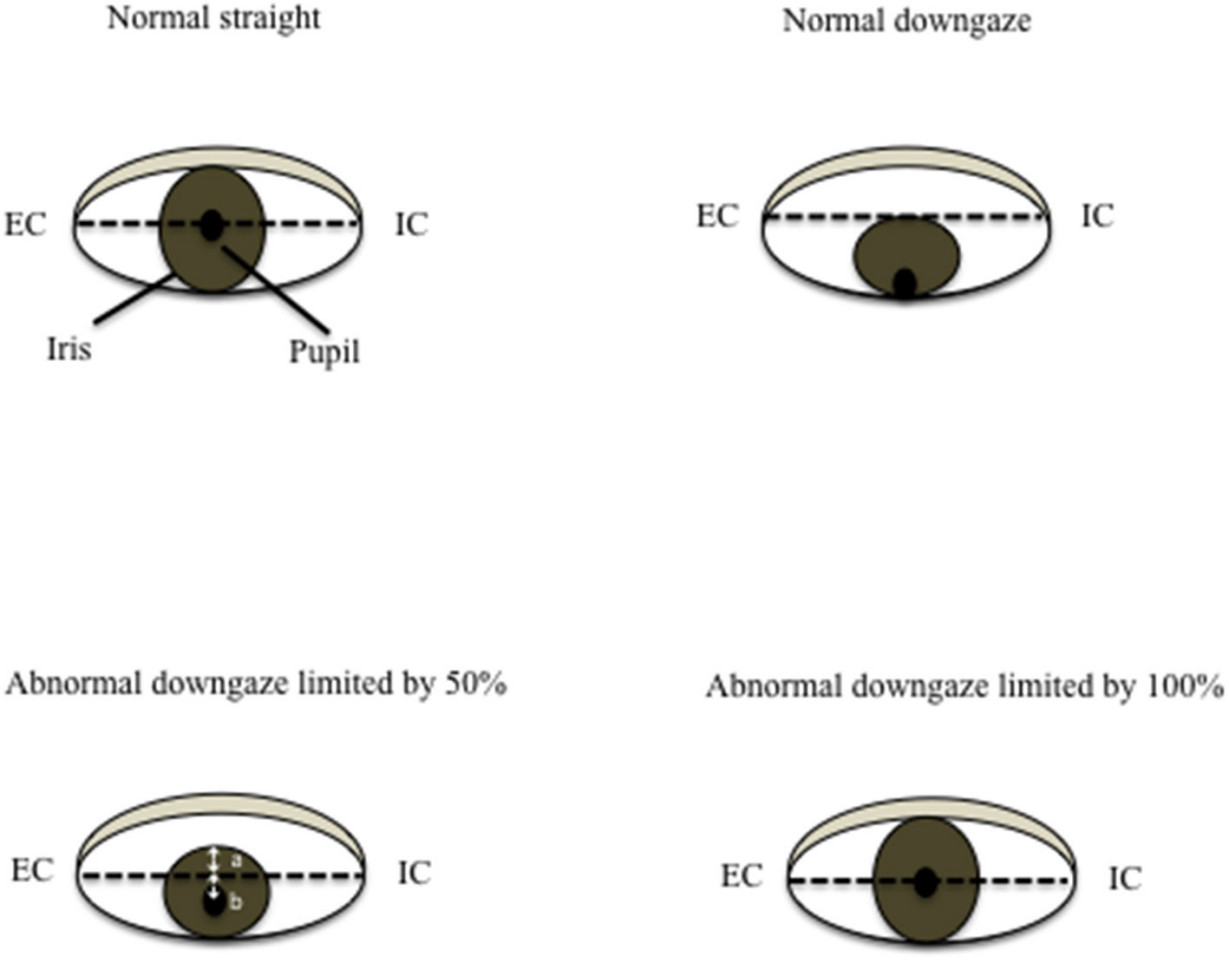
Measurement of downgaze palsy in principle. The severity of downgaze palsy is (a/r) × 100% or [a/(a+b)] × 100%. r, radius of the iris (r = a+b); EC, external canthus; IC, internal canthus.

**Figure 2:**
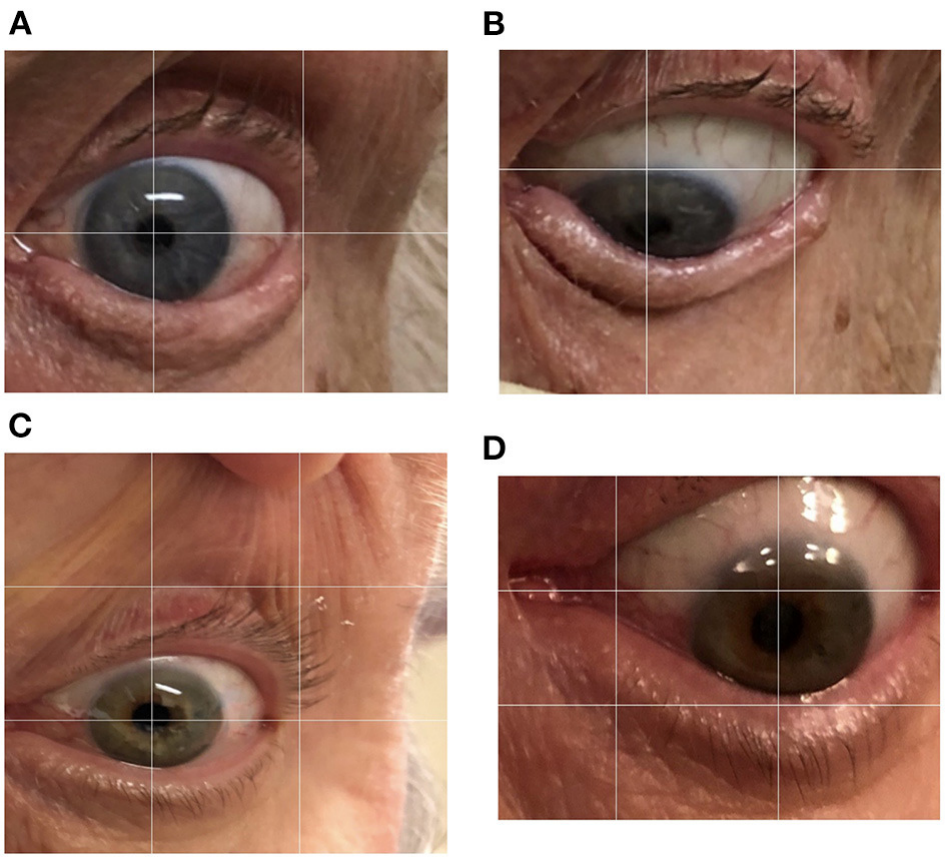
Quantification of downgaze palsy in practice. We can simply take a picture of the eyes using a common smart phone, and use the crop function to enlarge/rotate (if needed) the eye and superimpose a grid on it in the context of the intercanthal line, center of the pupil, and upper edge of the iris. To demonstrate the concept and bedside practice of this method, here we collected pictures showing a healthy eye in neutral position with a grid **(A)**, and in maximal downgaze with a grid **(B)**, an abnormal eye of a PSP patient's with 100% limited downgaze palsy with a grid **(C)**, and an abnormal eye of a PSP patient's with 70% limited downgaze palsy with a grid **(D)**.

In a healthy person without downgaze palsy, the center of the pupil should be in the middle of the intercanthal line (the line connecting the external canthus and internal canthus) in primary straight gaze, and the upper edge of the iris should be slightly below or just touching the intercanthal line when looking down as far as they can. The patient with 100% downgaze palsy is unable to look down at all (and unable to look up either at that stage, as upgaze is always first impaired). Thus, when this patient is asked to look downward as far as he/she can, the pupil (or the center of the iris) is in the middle of the intercanthal line. For others in between, we can obtain the percentage of downgaze palsy by dividing the portion of the iris above the intercanthal line (the distance between the upper edge of the iris and the intercanthal line, which we refer to as “a”) by the radius (r) of the iris (the distance between the upper edge of the iris and the center of the pupil, or the combination of measurement “a” and measurement “b” which stands for the measurement “below the inter canthus line” but above the center of the pupil) (Figure 1). In other words, the severity of downgaze palsy is expressed as (a/r) × 100% or [a/(a+b)] × 100% (Figure 1). We can simply take a picture of the eyes using a common smart phone, and use the crop function to enlarge/rotate (if needed) the eye and superimpose a grid on it in the context of the intercanthal line, center of the pupil, and upper edge of the iris. To demonstrate the concept and bedside practice of this method, here we collected pictures showing a healthy eye in neutral position with a grid (Figure 2A), and in maximal downgaze with a grid (Figure 2B), an abnormal eye of a PSP patient's with 100% limited downgaze palsy with a grid (Figure 2C), and an abnormal eye of a PSP patient's with 70% limited downgaze palsy with a grid (Figure 2D). The inter-rater agreement was assessed and found to reach 100% agreement between the movement disorder neurologist (TX) and the neuro-ophthalmologist (JN) if we were allowed a reasonable 10% difference in visual estimate (occurred in 2/6 tests) when we blindly tested at 0% limit, 15% (10–20%) limit, 30% limit, 60% limit, 75% (70–80%) limit, and 100% limit by visual estimate based on the simple calculation and principle as laid out as above. The 10% allowance in difference was based on the usual practice in rating scales, such as UPDRS and PSP rating scale (PSPRS), where each rating level contains a range of 15%–35% severity given the 5 rating levels of severity in the majority of the items (from normal level 0 to the worst level 4, such as tremor, bradykinesia, and rigidity for UPDRS, or oculomotor function for PSPRS). We also used Intraclass Correlation Coefficient (ICC) to evaluate the agreement on continuous data between two raters without the 10% allowance in difference, and found that the ICC is 0.98 with 95% confidence interval between 0.86 and 1.

### Statistical Analysis

Descriptive statistics for demographic study variables included mean with standard deviation (s.d.) and raw value with percentages for categorical variables. Linear regression was applied to all factors (including AO, sex, disease duration when the downgaze palsy was quantitatively rated, severity of the downgaze palsy quantitatively rated prospectively at that time as described above, medication use, UPDRS-III scores, and pulmonary and cardiovascular diseases) to identify factors and form an equation to estimate total survival duration. Overall survival (OS) was assessed using the Kaplan-Meier curve both as a whole cohort and by sex. Multivariate Cox models were also further used to evaluate the risk factors for survival duration, considering all the factors as above. All tests were two-sided with a *p*-value < 0.05 as statistically significant, and <0.1 as borderline significant. Analyses were performed using R software version 4.0.2.

## Results

### Basic Characteristics

We had 23 probable PSP-RS or PSP-P patients per MDS criteria (9), consisting of 9 women and 14 men. All patients had the hummingbird sign in sagittal MRI imaging when downgaze palsy was first documented, which further supported the diagnosis. All individual patient's information is listed in Table 1. AO (mean + s.d.) was 66.7 ± 7.9 years; disease duration when the first downgaze palsy was documented was 4.1 ± 3.4 years; the severity of downgaze palsy was 62.8 ± 31.2% then; the total survival duration from the disease onset to death was 6.6 ± 4.0 years; remaining survival duration after the assessment for the first downgaze palsy was 2.5 ± 1.4 years; cardiovascular disease was present in 17 patients (74%); pulmonary disease was present in 4 patients (17%); medication was tried in 10 patients (43%); UPDRS-III was recorded in all 23 patients, with an average score of 29.3 ± 9.1.

**Table 1:** Characteristics of the PSP patients.

**Subject**	**Sex**	**AO**	**DD**	**DGP**	**TSD**	**RSD**	**CVS**	**Pul**	**Med**	**UPDRS**
1	M	74	5	30	7	2	1	1	0	15
2	M	68	1.5	90	3	1.5	0	0	0	26
3	M	56	1	90	1.5	0.5	1	1	0	37
4	M	58	3.5	10	6.5	3	1	1	0	29
5	F	54	11	99	11.5	0.5	0	0	0	44
6	F	74	1	85	2.5	1.5	1	0	1	24
7	M	60	3	95	5.5	2.5	1	0	0	20
8	F	74	2	20	5	3	1	0	0	20
9	M	70	2	20	5	3	1	0	0	19
10	F	67	2	100	3	1	1	1	1	28
11	F	63	6	50	11.5	5.5	0	0	0	24
12	M	53	11	50	17	6	1	0	1	46
13	F	73	12	85	14	2	0	0	1	31
14	F	58	1.5	20	4.5	3	1	0	1	25
15	M	72	4.5	15	9.5	5	1	0	1	19
16	F	71	4	95	6	2	1	0	1	34
17	M	78	9	50	11	2	1	0	1	28
18	M	65	4.5	95	7	2.5	0	0	0	36
19	M	65	3	60	4.5	1.5	1	0	0	25
20	M	79	2	70	4	2	1	0	1	34
21	M	77	0.5	50	2.5	2	1	0	1	42
22	M	59	2.5	85	4.5	2	1	0	0	46
23	F	65	2.5	80	5	2.5	0	0	0	23

*AO, age at onset; DD, disease duration at assessment; DGP, downgaze palsy (%) then; TSD, total survival duration from onset of PSP to death; RSD, remaining survival duration after the assessment; CVS, cardiovascular diseases; Pul, pulmonary diseases; Med, medications; UPDRS, unified PD rating scale (motor section, part-III)*.

### Fitting the Total Survival Duration With Linear Regression

**Figure 3:**
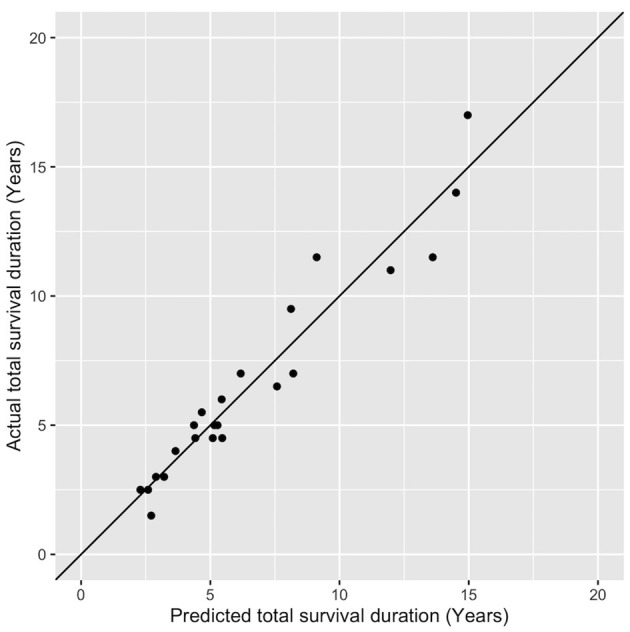
An accurate prediction of the total survival duration by using the equation compared to the actual values of the total survival duration. Each dot (formed by the predicted value in the *x*-axis and actual value in the *y*-axis) should be on the 45-degree diagonal line if the predicted value is equal to the actual value in the total survival year, which is virtually the case.

On linear regression, when all factors were considered, the factors significantly predicting the total survival duration were the disease duration (*p* < 0.001) and the severity of downgaze palsy at the first documentation (*p* < 0.05), with the AO as a borderline effect (*p* = 0.0715). We then further remodeled the data using these three factors and formed an equation to estimate total survival duration as below: 5.76 + (1.11 × DD)–(0.03 × severity of the DGP then) −0.03 × AO (adjusted *R*^2^ = 0.92), where the DD (disease duration) (*p* < 0.01) and the severity of DGP (downgaze palsy, ranging from 10–100 in applying the equation, as in Table 1) (*p* < 0.001) remained significant. We then tested the equation on the 23 cases in Table 1 and found an accurate fit to the total survival duration with a discrepancy of only 0.82 ± 0.67 years in the estimated values compared to the actual values of the total survival duration (Figure 3).

### Survival Analysis and Multivariate Cox Model for Survival Duration

**Figure 4:**
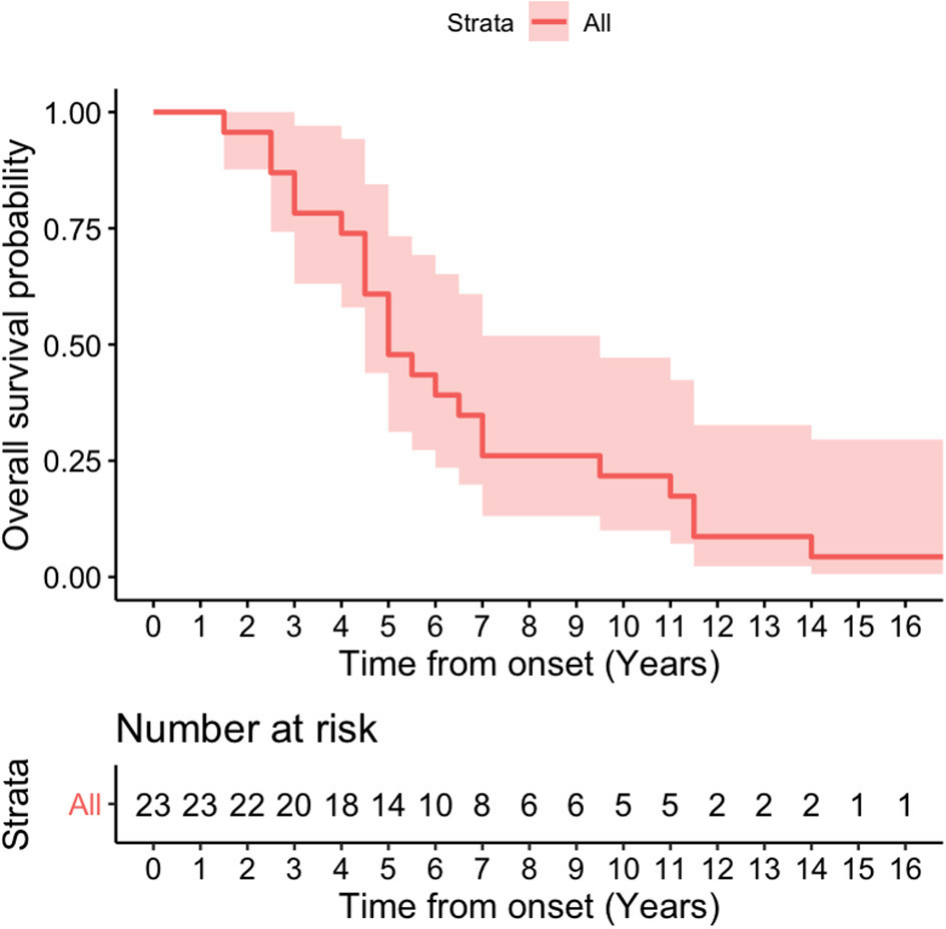
Kaplan-Meier survival curve in all patients. Overall median survival age from the onset to death was 5 years in this cohort.

**Figure 5:**
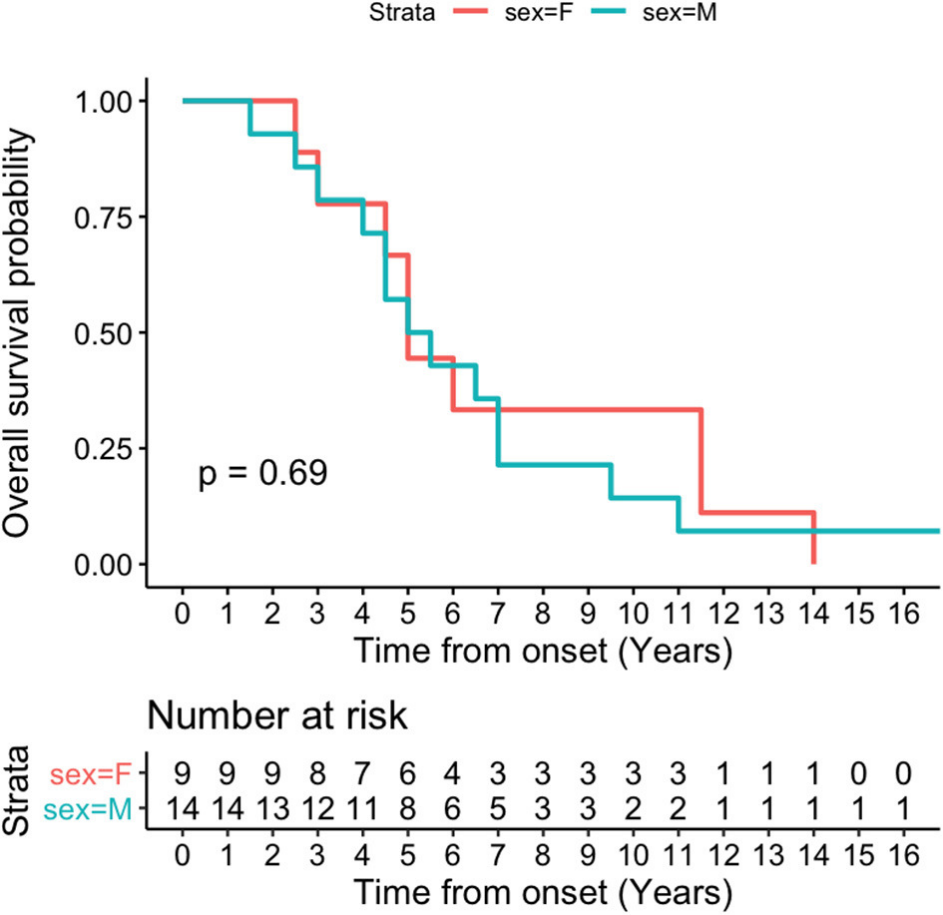
Kaplan-Meier survival curve in patients by sex. There is no difference on median survival age by sex in this cohort.

Kaplan-Meier survival curve was analyzed in all patients (Figure 4) and in patients according to sex (Figure 5). Overall median survival age from the onset to death was 5 years in this cohort (Figure 4). There was no difference in survival between the male and female PSP patients (Figure 5). Multivariate Cox model on total survival duration again found that the disease duration at the assessment when downgaze palsy was first quantitatively rated (exponential coefficient 0.3464, associated with long survival duration, *p* < 0.001) and severity of downgaze palsy then (exponential coefficient 1.0179, associated with short survival duration, *p* < 0.05) were the two significant contributing factors to the total survival duration, which was consistent with the conclusion from the linear regression analysis.

## Discussion

This is the first study to reveal that the severity of downgaze palsy when first noticed clinically and the disease duration at that time are the important factors to estimate the total survival duration in patients with probable PSP-RS or PSP-P, as concluded by the analysis from both the well-fitted linear regression model and the multivariate Cox model. We used both models to ensure confidence in the conclusion, as nobody was really sure which model was supposedly the best. The severity of downgaze palsy was prospectively and quantitatively rated based on a simple mathematical equation (a/r × 100%) measured at bedside. Based on this, we also further developed a regression equation to estimate the total survival duration [5.76 + (1.11 × DD)–(0.03 × severity of the DGP, ranging from 10 to 100, with 10 the least and 100 the worst palsy in percentage)–(0.03 × AO)] (adjusted *R*^2^ = 0.92), which yielded a very small discrepancy of only 0.82 ± 0.67 years in the estimated total survival duration compared to the actual total survival duration in this cohort. The remaining survival duration (estimated time to death after that assessment) could be easily inferred by subtracting the disease duration then from the estimated total survival duration. For example, for a 70-year-old patient presenting with disease duration of 5 years (AO at 65 years old) and downgaze palsy severity of 80 found during the exam (limited by 80% in downgaze amplitude), we could expect that this patient's total survival duration from the onset of PSP to death would be 7 years (5.76 + 1.11 × 5 −0.03 × 80–0.03 × 65 = 6.96). We could also expect that this patient unfortunately would most likely die 2 years after the visit (7–5 = 2).

Combining the severity of downgaze palsy with disease duration hence could allow us to predict prognosis (total survival duration) regardless of when and to what extent downgaze palsy is first documented or when the patient chooses to see the clinician leading to the discovery of downgaze palsy, as in real world clinical practice. The equation tells us that the occurrence of severe downgaze palsy soon after the onset of PSP (or a shorter latency of severe downgaze palsy) is associated with worse prognosis or shorter total survival duration, particularly in an older patient, and vice versa. It also is consistent with the observation that patients with PSP-RS (who usually have shorter latency and more severe downgaze palsy) have shorter survival compared to patients with PSP-P (who usually have longer latency and milder or no downgaze palsy) (5, 6).

This study has significant clinical applications. We can use a simple bedside exam to predict the total survival duration or the remaining duration of the disease in minutes. This can help clinical counseling of patients and their families and clinical management as well. Moreover, this can also allow us to correctly stage our patients for clinical trial enrollment, where an accurate estimation of the remaining survival duration is important for the long duration PSP trials (10).

It is worth noting that it is not uncommon in clinical practice to encounter PSP patients with blepharospasm, apraxia of eyelid opening, or light sensitivity, which could seemingly pose challenges to an eye exam. However, none of the eye conditions should affect the measurement as we usually hold up the patient's upper eyelids in order to clearly see the extent of the downgaze palsy. We could further overcome the patient's light sensitivity by dimming the light in the exam room and giving the patient a bit more time to accommodate.

Our previous study highlighted the importance of the latency of downgaze palsy as a predicting factor for survival duration (5), while our current study is more applicable for use in the real world by combining the severity of downgaze palsy and disease duration regardless of when patients are first evaluated by a clinician to accurately estimate survival. Other potential confounding factors, such as sex, medication use for parkinsonism, parkinsonism severity, pulmonary diseases, and cardiovascular diseases, were also explored, but none were found to significantly affect the total survival duration. Sex was also not found to be associated with prognosis in a different study (8). Medication status was not expected to be an important factor, as PSP usually does not respond well to medications for parkinsonism. Parkinsonism was not found to be a key factor in total survival duration, likely because the UPDRS-III does not include some PSP-specific symptoms related to death, such as the swallowing dysfunction. Our study is plausible from the pathological and clinical standpoint, given the fact that downgaze palsy is the most specific feature of PSP (1), which is not related to aging (2), but controlled by the fibers in the STN, GPi, SN, midbrain, and frontal and parietal cortexes (3), along the path where PSP pathology is formed and spreads (4). Hence, downgaze palsy is expected to serve as a reliable marker to predict survival duration. We only included patients who died of causes attributable to death related to PSP, and excluded those who died of causes unrelated, such as cancer, stroke, and motor vehicle accident, in order to make sure that the study reflects the natural disease course. Our patients have similar AO, age at death, and survival duration to PSP populations reported elsewhere, which suggests that our patient population is generally similar to others (5, 6, 8).

There are some limitations in this study. First, the sample size is small, as PSP is a relatively rare disease. Second, the diagnosis was not autopsy verified, although these patients were probable PSP (-RS or -P), the highest confidence level we can achieve without autopsy, and they all had typical hummingbird sign, which is highly specific and supportive for PSP (11). Third, the study is lacking machinery measurement of downgaze palsy. The equipment to make this measurement in PSP patients in a clinical setting is not commercially available to our knowledge. We have a quantitative measurement of downgaze by a simple bedside mathematical equation for visual estimation, with a high inter-rater agreement between the movement disorder specialist and the neuro-ophthalmologist. Various non-machinery clinical scales have been widely used in this field, and the measurement of downgaze palsy is added here as another one. Fourth, we do not have correlation data between PSPRS score and prognosis due to the lack of sufficient data on PSPRS when downgaze palsy was measured. Fifth, we do not have swallowing dysfunction data in this analysis. Sixth, our data may only be applicable to patients with PSP-RS and PSP-P, as the pathology on other types may not follow the typical path as mentioned (12). For example, the PSP-CBS may start with the parietal lobe cortex before it involves the basal ganglia and substantia nigra. However, PSP-RS and -P together represent 86% of the PSP patients (6), which makes our results encompass the majority of PSP patients with significant practical application. Seventh, slow saccade could be a more sensitive measurement than downgaze palsy for the diagnosis of PSP. However, slow saccades are not specific for PSP and difficult to quantify, while downgaze palsy is very specific for PSP (1). There is a wide range on the onset of downgaze palsy in PSP (5), with PSP-RS usually presenting with downgaze palsy much earlier than PSP-P (1, 5, 6). The average survival duration is 5.9 years in PSP-RS and 9.1 years in PSP-P (6), which suggests the need to have personalized measurements to estimate survival duration in individual patients to help clinical practice and clinical trials.

Although the role of the quantitative downgaze palsy in the context of the disease duration on individual survival has not been explored before, the effects of other factors on survival in general were studied in the literature. Golbe et al. reported that the UPSPRS score could estimate the probability of the survival (13). Litvan et al. reported that onset of falls during the first year, early dysphagia and incontinence predicted a shorter survival (8). PSP-RS type, male gender, older age of onset, and a short interval from disease onset to reaching the first clinical milestone of frequent falling, cognitive disability, unintelligible speech, severe dysphagia, dependence on wheelchair for mobility, the use of urinary catheters, and placement in residential care were all independent predictors of shorter disease duration to death (14). PSP-RS type, early development of constipation, and urinary symptoms were also associated with higher risk of reaching the first disease milestone and with a shorter survival in these patients (15). Similarly, older age at onset, early falls, speech and swallowing problems, diplopia, and early insertion of the percutaneous gastrostomy predicted reduced survival (16). PSP-RS type, early dysphagia, and early cognitive symptoms were associated with short survival (17). Xie et al. also reported that early cognitive dysfunction and incontinence predicted a shorter survival (5). Higher serum neurofilament light chain levels were also found to be associated with a shorter survival (18). Cerebrospinal fluid neurofilament light chain and tau protein were found as mortality biomarkers in parkinsonism, including PSP, but it was unable to tell the difference among each specific parkinsonian type (19). The LRRK2 gene was associated with reduced survival (20). Atrophy in midbrain and larger third ventricle width were also associated with higher risk of death (21, 22). We were unable to control all the factors in our study revealing that the rapid deterioration of the downgaze palsy could predict survival in an individual level, although we controlled other factors and also consistently found that older age of onset was also associated with a shorter disease duration to death.

In summary, in this prospective survival study, we identified contributing factors and formed a novel bedside equation to estimate the total survival duration (or remaining survival duration as subsequently inferred) in patients with probable PSP-RS or PSP-P with highly specific hummingbird sign on brain MRI. Severity and time to develop downgaze palsy could help to predict the survival duration in patients with the major forms of PSP. These results might fulfill unmet needs in clinical practice to assist with patient/family counseling and clinical management and to identify the accurate stage of patients being considered for clinical trials with long duration of follow up. A further study with a larger cohort and more factors to control is needed to corroborate our conclusion.

## Data Availability Statement

The original contributions presented in the study are included in the article/supplementary material, further inquiries can be directed to the corresponding author/s.

## Ethics Statement

The studies involving human participants were reviewed and approved by The University of Chicago IRB. The patients/participants provided their written informed consent to participate in this study.

## Author Contributions

TX: study concept, data collection, analysis, interpretation, manuscript drafting, revision, and final approval. CY: data collection, manuscript revision, and final approval. WK: data analysis, manuscript revision, and final approval. MP, TH, and JN: manuscript revision and final approval. All authors contributed to the article and approved the submitted version.

## Conflict of Interest

TX has been supported in research by the Michael J Fox Foundation for Parkinson's Research and Parkinson's Foundation, and received honoraria from CVS Caremark for consultation and from Parkinson's Foundation for study sessions. The remaining authors declare that the research was conducted in the absence of any commercial or financial relationships that could be construed as a potential conflict of interest.

## Publisher's Note

All claims expressed in this article are solely those of the authors and do not necessarily represent those of their affiliated organizations, or those of the publisher, the editors and the reviewers. Any product that may be evaluated in this article, or claim that may be made by its manufacturer, is not guaranteed or endorsed by the publisher.
